# Rheostat Re-Wired: Alternative Hypotheses for the Control of Thioredoxin Reduction Potentials

**DOI:** 10.1371/journal.pone.0122466

**Published:** 2015-04-13

**Authors:** Kathryn D. Bewley, Mishtu Dey, Rebekah E. Bjork, Sangha Mitra, Sarah E. Chobot, Catherine L. Drennan, Sean J. Elliott

**Affiliations:** 1 Department of Chemistry, Boston University, Boston, Massachusetts, United States of America; 2 Howard Hughes Medical Institute, Cambridge, Massachusetts, United States of America; 3 Department of Chemistry, Massachusetts Institute of Technology, Cambridge, Massachusetts, United States of America; 4 Department of Biology, Massachusetts Institute of Technology, Cambridge, Massachusetts, United States of America; Instituto de Biociencias—Universidade de São Paulo, BRAZIL

## Abstract

Thioredoxins are small soluble proteins that contain a redox-active disulfide (CXXC). These disulfides are tuned to oxidizing or reducing potentials depending on the function of the thioredoxin within the cell. The mechanism by which the potential is tuned has been controversial, with two main hypotheses: first, that redox potential (*E_m_*) is specifically governed by a molecular ‘rheostat’—the XX amino acids, which influence the Cys pK_a_ values, and thereby, *E_m_*; and second, the overall thermodynamics of protein folding stability regulates the potential. Here, we use protein film voltammetry (PFV) to measure the pH dependence of the redox potentials of a series of wild-type and mutant archaeal Trxs, PFV and glutathionine-equilibrium to corroborate the measured potentials, the fluorescence probe BADAN to measure pK_a_ values, guanidinium-based denaturation to measure protein unfolding, and X-ray crystallography to provide a structural basis for our functional analyses. We find that when these archaeal thioredoxins are probed directly using PFV, both the high and low potential thioredoxins display consistent 2H^+^:2e^-^ coupling over a physiological pH range, in conflict with the conventional ‘rheostat’ model. Instead, folding measurements reveals an excellent correlation to reduction potentials, supporting the second hypothesis and revealing the molecular mechanism of reduction potential control in the ubiquitous Trx family.

## Introduction

Thioredoxins and thioredoxin-like proteins (glutaredoxins and protein disulfide isomerases) are prevalent in nature and found throughout all kingdoms of life [1]. These disulfide/dithiol oxidoreductases function via their surface-exposed, redox-active disulfide bond. The cellular functions of these proteins vary, ranging from electron transfer in response to oxidative stress to folding and refolding of proteins [1,2], while their varied functions are linked to the range of redox potentials spanned by the disulfide bond, nearly 300 mV [3,4]. The oxidative proteins such as DsbA and protein disulfide isomerase (PDI), aid in protein folding and have disulfide bonds with high redox potentials: reported values range from -89 mV to -124 mV [5–8] and -110 mV to -190 mV [9,10], respectively. Classical thioredoxins, such as Trx1 from *E*. *coli*, possess disulfide bonds of low redox potentials (-270 mV [11]), and have the ability to donate electrons to its partner proteins, one being ribonucleotide reductase [12].

**Fig 1:**
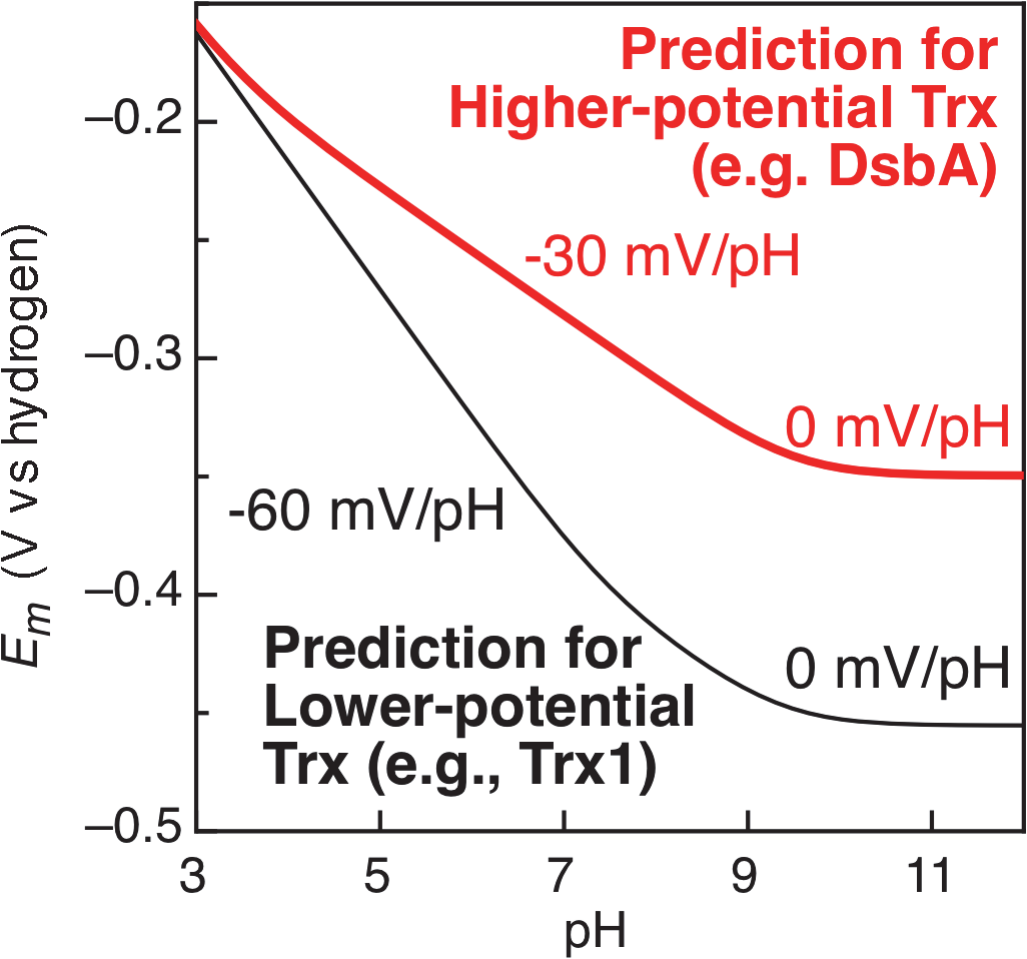
Theoretical and Experimental Pourbaix diagrams for DsbA and *E. coli* Trx1. Using the Nernst equation and the cysteine pKa values of DsbA (3.5) and Trx1 (7.1), the calculated dependence of redox potential on pH yields a difference in redox potential that is reflected in the different slopes within the Pourbaix diagram for higher-potential proteins (red) and lower-potential Trx proteins (black).

One of the major questions that arises from studying thioredoxins, is why are the disulfide bonds of some members of this superfamily “oxidizing” and others “reducing”? What inherently governs the redox potential of the disulfide/dithiol redox couple? Understanding the molecular basis for these differences has been a topic of ongoing research for several decades [4–6,8,9,11,13–17] with most studies focusing on the *E*. *coli* proteins Trx1 and DsbA and their various mutants [4–6,11,13–16]. From the work presented to date, there are two prevalent hypotheses. The first is that the identity of the CXXC motif and the pK_a_ of the N-terminal cysteine thiol largely govern the redox potential of the disulfide bond, as a redox-based ‘rheostat’ [4,5,15]. In high potential DsbA, for example, the CXXC active site contains a histidine residue that has been proposed to hydrogen bond to the N-terminal cysteine in the reduced form of the protein [17], causing the pKa of that cysteine to shift to a lower value (~3.5) as has been proposed from spectroscopic measurements [15,16,18]. In contrast, lower potential (or, “reducing”) Trxs lack a histidine in the CXXC motif and the spectroscopically observed cysteine thiol pK_a_ values are typical (~8) [19]. According to the pK_a_ model [4], the redox potential for the disulfide bonds found in both higher- and lower-potential Trx should be the same at infinitely low pH and then diverge in the physiological pH range due to the action of the redox linked pK_a_ values, resulting in the theoretical Pourbaix diagram shown in Fig 1 [4]. In particular, divergence would occur above pH 3.5 (the proposed unique pK_a_ of DsbA), as the unique pK_a_ associated with a higher-potential Trx redox couple yields a 1H^+^:2e^−^ process (with a slope of -30 mV/pH). In contrast, the Trx proteins with a lower-potential disulfide bond follow a 2H^+^:2e^−^ redox reaction [20] with slope of -59 mV/pH (25°C), due to the proposed similarity of the two Cys pK_a_ values. At high pH values (~9), the slope becomes flat (0 mV/pH), due to the fact that both cysteines would now be protonated. However, this calculated model is supported by experimental redox potential data collected only at physiological pH [4]. Additionally, the theory-based model relies on pK_a_ values determined by solution measurements, which themselves are not self-consistent [21]. (Indeed, the sparse data available on the pH dependencies of redox potentials for DsbA itself do not reveal a clear break in a Pourbaix diagram [5],[6], and suggest a slope of ~ -49 mV/pH, between the two limits of the pK_a_-based model). Here for the first time we compare complete data sets of pH dependent redox potentials for high and low potential disulfide bond containing proteins using direct electrochemistry, and show that solution-based, spectroscopically determined pK_a_ measurements are not relevant for predicting redox potential.

The second hypothesis suggests that the overall fold of the protein dictates whether the oxidized or the reduced form is more stable thermodynamically, and thus influences the redox potential of the disulfide bond [5,6,22,23]. This view has been validated for DsbA, which is thought to undergo a global conformational change upon reduction where the oxidized state is thought to be ‘tense’ and the reduced state is more stable [6], and data consistent with this mechanism is available for *E*. *coli* Trx1, which is thought to only undergo local active site conformational changes [24]. In both wild-type and CXXC mutants of *E*. *coli* Trx1 [13], this second hypothesis is able to predict the redox potential found experimentally at pH 7. However, no series of Trx proteins has been examined in a side-by-side comparison of pK_a_-based versus folding-based models.

In this paper we systematically test both hypotheses with a series of thioredoxins from *Archaeoglobus fulgidus (Af)* and *Thermoplasma acidophilum (Ta)*, which have been shown previously to possess disulfide bond redox potentials spanning -32 mV to -287 mV at pH 6 [3]. We have created various mutants installing hypothesized high-potential, histidine containing XX motifs into low-potential Trx protein folds. Our data include a thorough electrochemical study, cysteine pKa values, unfolding thermodynamic parameters, as well as crystallographic data showing that the archaeal Trx used for the basis of mutagenesis possesses a structure that is globally identical to other canonical Trx proteins. Together, these data show that the spectroscopically determined pK_a_ values of Cys residues in the archaeal proteins do not correlate with redox potentials, which is further corroborated by Pourbaix diagrams of both oxidizing and reducing-Trx proteins. In contrast, global unfolding energies yield an excellent correlation with reduction potential, suggesting that the CXXC ‘rheostat’ may well be governed more by folding than a specific pK_a_-based ‘wiring’.

## Materials and Methods

### Mutagenesis, Protein Expression and Purification

The expression and purification of *Af*Trx1, *Af*Trx3 [3] and *Ta*Trx [25] have been described previously. The *Af*Trx3 mutants were created using a mutagenesis kit (Stratagene) and the primers listed in **S1 Table**. The mutations were confirmed by DNA sequencing (Genewiz). The mutants were prepared in a similar manner to the wild-type protein. The empty pET-39b(+) vector (Novagen) was used as the source of DsbA. The pET-39b(+) vector was modified by adding a stop codon at the end of the C-terminal His-Tag. This modification allowed DsbA to be purified with a His-Tag, but removed 73 extraneous amino acids. DsbA was then expressed and purified using the same conditions as *Af*Trx3. The following two primers were used to install the stop codon: forward 5’—C CAT CAC CAT CAC TGA GCG GGT CTG GTG—3’ and reverse 5’- CAC CAG ACC CGC TCA GTG ATG GTG ATG G—3’.

### Protein Film Voltammetry

All protein film voltammetry experiments were conducted with a PGSTAT 12 potentiostat (Ecochemie) housed in a Faraday cage. A three electrode configuration was used and the temperature was controlled by using a circulator and a water-jacketed cell. The setup included a platinum counter electrode, a calomel reference electrode, and a pyrolytic graphite edge (PGE) working electrode. The PGE electrodes were sanded, polished with 1 μm alumina and sonicated before use. Protein films were generated either by directly depositing a concentrated solution of protein on the electrode, by soaking the electrode overnight in a dilute protein solution, or by spin depositing. Spin deposition was achieved by rotating the electrode (200 rpm) in a dilute solution of protein while applying either no voltage, or by scanning over a range of potentials. Experiments were performed using argon purged buffer (20 mM NaAc, HEPES, MES, TAPS, CHES, 50 mM NaCl) at 10°C. The raw data from each experiment was baseline subtracted and analyzed using SOAS [26].

### Glutathione Equilibrium Measurements

The redox equilibrium between glutathione and thioredoxin [5,9] was used to corroborate the midpoint potential of *Af*Trx3, *Af*Trx3HP and *Af*Trx3PH, assuming a reference potential of the GSSG/(GSH)^2^ redox couple of -240 mV. Thioredoxin (1–2 μM) was equilibrated with 10 μM oxidized glutathione (GSSG, Sigma Aldrich) and varying amounts of reduced glutathione (0–200 mM, GSH, Sigma Aldrich) in argon purged buffer (100 mM sodium phosphate, pH 7 with 1 mM EDTA). The reactions were allowed to equilibrate in an MBraun glovebox overnight. A fluorescence spectrum was taken for each sample (280 nm excitation, 300–400 nm emission, 25°C). The intensity at 345 nm was plot against [GSH]^2^/[GSSG] to obtain K_eq_, which was used to calculate the midpoint potential of thioredoxin as in References [5,9].

### Cysteine Thiol pK_a_ Determination

The method to determine the cysteine thiol pKa was adapted from Lewin *et al* [27,28]. Reduction of Trx was achieved with a 20-fold excess of DTT. After incubation for 30 minutes the excess DTT was removed with a PD-10 column. A mixed buffer system of 50 mM Tris HCl, sodium citrate, K_2_HPO_4_ and CHES or 50 mM KCl, acetate, MES and Tris HCl was used for the assay. Identical results were obtained from either buffer system. The fluorescent probe BADAN [6-bromoacetyl-2-dimethylaminonaphthalene] (AnaSpec) was made fresh in DMF every 45 minutes, as the intrinsic fluorescence intensity decreased after this amount of time. Reduced Trx (0.25 μM) was allowed to equilibrate with buffer of various pH values at 23°C. Kinetic traces were then obtained by adding BADAN (3.25 μM), and recording the fluorescence emission at 536 nm (excitation wavelength 387 nm) using a SpectraMax M2 plate reader (Molecular Devices). The initial rate (linear slope) of the reaction was obtained and was plot against pH. It was found that similar results could be obtained by plotting the initial fluorescence intensity (when an equal amount of BADAN and Trx were used) verses pH.

### Protein Unfolding/Refolding Fluorescence

A stock solution of 8 M guanidine hydrochloride (Sigma Aldrich), pH 7 was used in the unfolding experiments. By using varying amounts of guanidine and buffer (10 mM HEPES, 150 mM NaCl, pH 7) the protein (final concentration of 5 μM) was equilibrated in 0 M—7 M guanidine HCl (GdnHCl) for 24–72 hours at room temperature. Oxidized unfolding experiments were performed with air-oxidized protein. For the reduced unfolding experiments, protein samples were pre-reduced with dithiothreitol (DTT), and a final concentration of 1 mM DTT was added to each protein/guanidine solution to keep the protein in its reduced state. The reduced samples were also prepared and allowed to equilibrate an MBraun glovebox.

A Horiba Jobin Yvon FluoroMax 3 fluorimeter was used to collect the fluorescence unfolding data, using a 5 mm x 5 mm path length quartz cuvette, thermostated at 25°C with a water circulator. An excitation wavelength of 280 nm was used with a 2 mm slit width, and emission spectra were collected at 300–450 nm (3 mm slit width). Data was taken every 1 nm with a 0.5 sec integration time.

### Protein Crystallization and X-Ray Crystallography

*Af*Trx3HP crystals were grown at room temperature by incubating 1.0 μl of 20 mg/ml protein solution (in 10 mM HEPES pH 7.0 and 20 mM NaCl) and 1.0 μl of precipitant solution containing 60% Tacsimate, pH 7.0 using sitting drop method. Rod-shaped crystals of approximately 100–150 μm grew in 3–4 days. Crystals were cryo-protected in precipitant solution containing 10% glycerol by soaking for 2–5 minutes before flash-freezing in liquid nitrogen.

X-ray diffraction data were collected at 100 K in-house using a Rigaku R-AXIS IV IP detector. Data were subsequently integrated and scaled in DENZO and SCALEPACK respectively [29]. The crystals belong to the orthorhombic space group P2_1_2_1_2_1_, with two thioredoxin molecules per asymmetric unit. The structure was solved to 1.95 Å resolution by molecular replacement using *Staphylococcus aureus* thioredoxin (PDB ID 2O7K) as a search model. Water molecules were removed from the initial search model used for molecular replacement in PHASER [30] that gave a Z-score of 12.0, suggesting a correct solution. Using a chainsaw model of the initial solution, correct residues and side chains were introduced in COOT [31] and a restrained refinement in REFMAC [32] from the CCP4 Program Suite [33] resulted in R_*free*_ of 35.0% and R_*work*_ of 29.9%. Subsequent rounds of refinement were carried out in CNS [34], which included iterative rounds of energy minimization, B-factor refinement, simulated annealing. Final refinement was carried out in PHENIX [35], which allowed Cys 59 and Cys 62 to be refined as a 50:50 mixture of oxidized and reduced disulfide bonds. Model building was performed in COOT using SigmaA weighted 2F_o_-F_c_ and F_o_-F_c_ maps. Water molecules were included automatically using COOT and were manually checked against 2F_o_-F_c_ and F_o_-F_c_ electron density maps. The structure was analyzed using 2F_o_-F_c_ composite omit maps and Ramachandran geometries were analyzed with PROCHECK [36]. The final model contains residues 25–134 (of 134) for chain A and residues 26–134 (of 134) for chain B (Protein Data Bank (PDB) ID 4XHM.pdb).

## Results

### Re-wiring the ‘rheostat’: *Af*Trx is structurally homologous to canonical Trx

Mutants of *Af*Trx3 that altered the identity of the residues between the two active site cysteines were generated (**Table 1**) to test the CXXC redox rheostat model, where hydrogen bonding between the N-terminal Cys and a histidine within this active site is proposed to occur [17]. We therefore created mutants of the wild-type CMPC sequence, substituting histidine, as well as lysine at the XX positions. As the names imply, the mutant *Af*Trx3HP has the active site sequence CHPC; *Af*Trx3PH has the sequence CPHC, and *Af*Trx3KP has the sequence CKPC (Table 1). These were successfully expressed and purified.

**Table 1:** Electrochemical potentials and Cys pKa values of thioredoxins.

Trx	CXXC	E_m_ (mV)	δ (mV)	Spectroscopic Cys pK_a_ value
*Ta*Trx	CHPC	-64^a^	60	6.5 ± 0.1
*Af*Trx1	CPHC	-32^b^	62^b^	6.8 ± 0.1
*Af*Trx3	CMPC	-287^b^	56^b^	7.0 ± 0.1
*Af*Trx3HP	CHPC	-291^a^	56	7.0 ± 0.1
*Af*Trx3PH	CPHC	-221^a^	60	4.2 ±0.2, 7.3 ± 0.3
*Af*Trx3KP	CKPC	-315^a^	46	7.2 ± 0.1
DsbA	CPHC	-89^c^,-124^d^	—	3.1 ± 0.2

^a^determined by PFV at pH 7.0

^b^from Ref [3]

^c^from reference [5]

^d^ from Ref [6–8].

**Fig 2:**
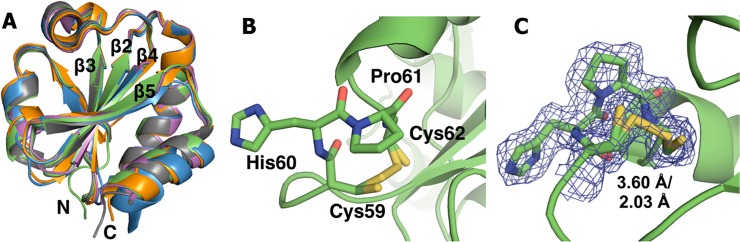
Structural analysis of thioredoxins. (A) Structural comparison of *Af*Trx3HP (green) with thioredoxins from *S*. *aureus* (magenta), *E*. *coli* (orange), *T*. *thermophilus* (grey), and *A*. *acidocaldarius* (sky blue) with r.m.s.d’s of 0.66 Å, 0.68 Å, 0.40 Å, and 0.70 Å respectively. (B) *Af*Trx3HP cysteine loop with the active site CHPC motif. (C) 2F_o_-F_c_ α_omit_ density (contoured at 1 σ) is consistent with 50:50 mixture of oxidized (2.03 Å distance) and reduced (3.60 Å distance) conformations of the Cys 59-Cys 62 disulfide. The main chain and side chain residues are shown as sticks with oxygens in red, nitrogens in blue, sulfurs in yellow, and carbons are in green similar to protein backbone.

To ensure that *Af*Trx3 is a representative Trx, the crystal structure of *Af*Trx3HP was solved to 1.95 Å resolution (see **S2 Table**). The *Af*Trx3HP mutant crystallized better than wild-type and thus was used to provide a structural depiction of this archaeal protein. The structure consists of two molecules in the asymmetric unit (a.s.u), where both structures are complete except for the first 25–26 residues at N-termini, which are disordered. *Af*Trx3HP shares the classic Trx fold, consisting of a central core of five β-strands enclosed by four α-helices (**Fig 2A**). The active site loop is found at the end of a α-helix on the surface of the protein where it can interact with its partner proteins to catalyze disulfide exchange. In both structures in the a.s.u., the Cys 59-Cys 62 disulfide bond appears to be a ~50:50 mixture of oxidized and reduced conformations (**Fig 2B** and **2C**). Comparison with *E*. *coli* Trx in the region of the active site shows that the only major difference is in the sequence of the CXXC loop itself. In particular, the difference is due to the substitution of His (60 in this *A*. *fulgidus* mutant) with Gly (33 in *E*. *coli*). All other residues near the active site disulfide are conserved (see below).

### Protein Film Voltammetry Reveals 2H^+^:2e^−^ Stoichiometries

**Fig 3:**
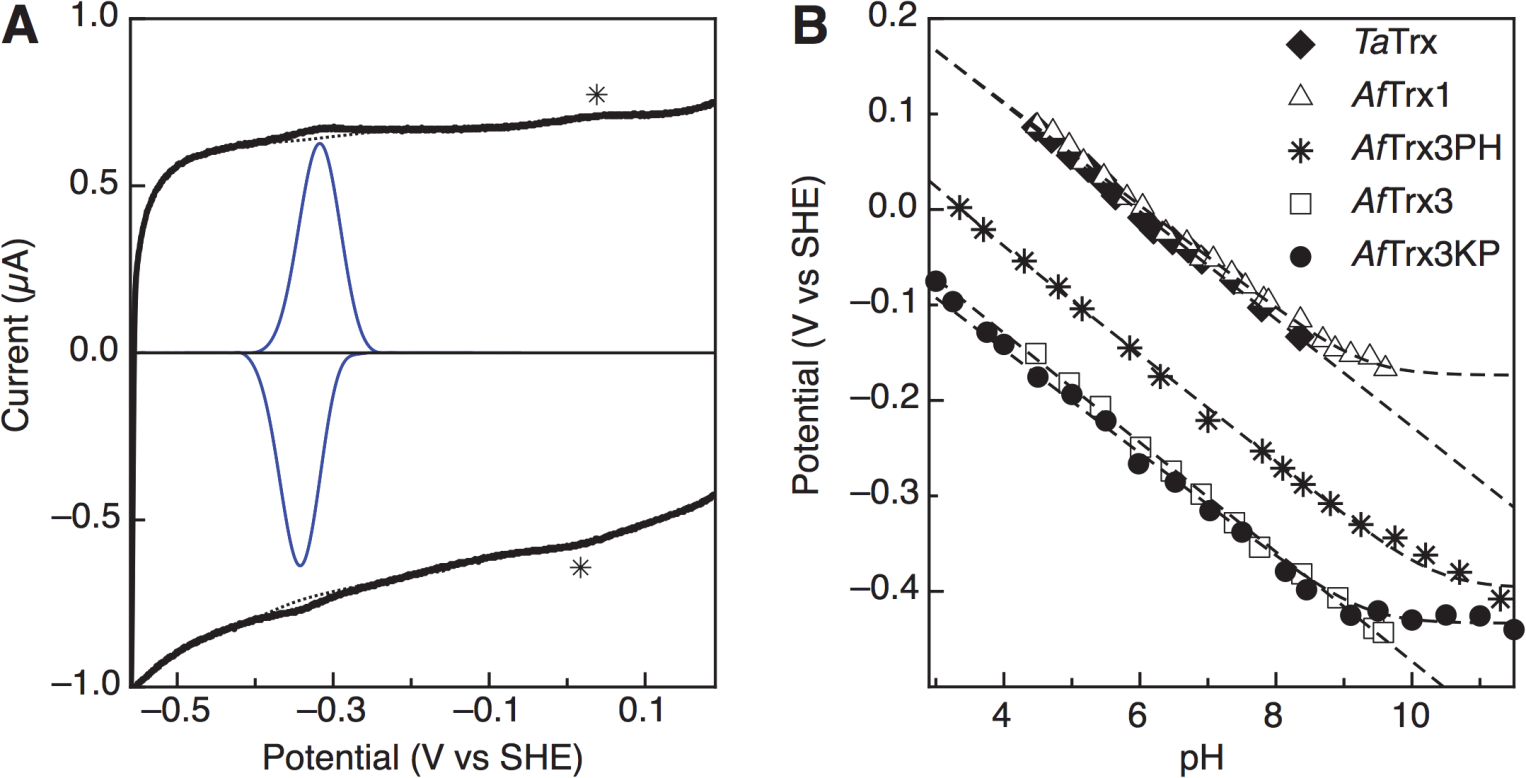
Direct electrochemistry of archaeal Trx proteins. (A) An example of direct PFV analysis of Trx3s proteins. Background capacitance of the blank electrode (dashed line) can be subtracted from the raw data (heavy) to reveal highly cooperative 2-electron redox couples (solid line), with a surface feature of graphite electrodes indicated as *. (B) Pourbaix diagrams for thioredoxins *Ta*Trx (diamonds), *Af*Trx1(triangles), *Af*Trx3PH (asterisks), *Af*Trx3 (squares), and *Af*Trx3KP(bullets).

To assess the possible role of redox-linked pK_a_ values in distinguishing Trx disulfide bonds of varying potential, we conducted protein film voltammetry (PFV) studies of the Trx proteins and mutants of *Af* Trx3. Direct electrochemistry of these proteins allows for the precise evaluation of the redox couple of the disulfide bond itself through a cyclic voltammetry experiment of an immobilized protein sub-monolayer (**Fig 3A**). This approach allows for the facile determination of the pH dependence of *E*_*m*_, plotted as a Pourbaix diagram (**Fig 3B)** that allows for comparison of novel Trx proteins studied here with our prior results for *Af* Trx3 [3].

The Trx proteins studied here have a range of *E*_*m*_ values at pH 7, which is loosely correlated to the identity of the variable residues in the CXXC motif (**Table 1**), where the uncertainty is 2–3 mV for each Trx examined. *Ta*Trx displays a high potential (similar to *Af*Trx1) and both possess a His residue in the variable region of the CXXC motif. However, the *Af*Trx3PH mutant, which also has a His residue in the CXXC motif, has a midpoint potential that is only slightly higher than wild-type *Af*Trx3, which has no His. At neutral pH the other mutants that have installed alternative residues in the CXXC motif of the *Af*Trx3 active site were found to be similar to wild-type (*e*.*g*., *Af*Trx3HP and *Af*Trx3KP proteins). Regardless of their redox potential at pH 7, Pourbiax diagrams reveal parallel slopes of approximately -56 mV / pH unit, the theoretical value for a 2H^+^:2e^-^ coupled process over the entirety of the pH range studied. Critically, there does not appear to be a pK_a_ value observed between pH values of 3 and 9, as predicted by the supposition that a high-potential disulfide bond results from a uniquely low Cys pK_a_ in the Trx ‘rheostat’. Only *Af*Trx1, *Af*Trx3PH and *Af*Trx3KP show pK_a_ values that can be determined from electrochemistry: 9.1, 10.4, and 9.2, respectively. Although it is clear that the pK_a_ values must be nearly identical for both Cys residues for *Ta*Trx and *Af*Trx3, the precise pK_a_ values could not be measured as the electrode:protein interaction was not stable at high pH values.

Analysis of the voltammetric peak width at half-height reveals the number of electrons giving rise to that signal. Although the theoretical values of 1- versus 2-electron processes are 86 mV and 43 mV, respectively, we have previously shown that the peak widths of thermophilic thioredoxins on graphite electrodes are artificially broadened, to ~60 mV at the low temperature of 10°C [3], which is used to ensure stability of the protein film over multiple experiments. Consistent with our prior findings of disulfide-bond electrochemistry, these Trxs show peak widths around 60 mV at 10°C (**Table 1**), indicating 2-electron processes.

To corroborate the shifts in redox potential for the *Af*Trx3 mutants, glutathione (GSH/GSSG) redox titrations were used at pH 7. In all cases, the titration results agreed with PFV, though the measured potentials were slightly shifted to more oxidizing potentials: The variations range from 9 mV more positive (for *Af*Trx3PH, where the *E*_*m*_ value measured by PFV was -221 mV, compared to -212 mV from titrations) to 20 mV more positive (for *Af*Trx3, where *E*_*m*_ was -287 mV by PFV, and measured as -267 by titration).

### Higher-potential Disulfide Bonds Need Not Have Low Cys pK_a_ Values

**Fig 4:**
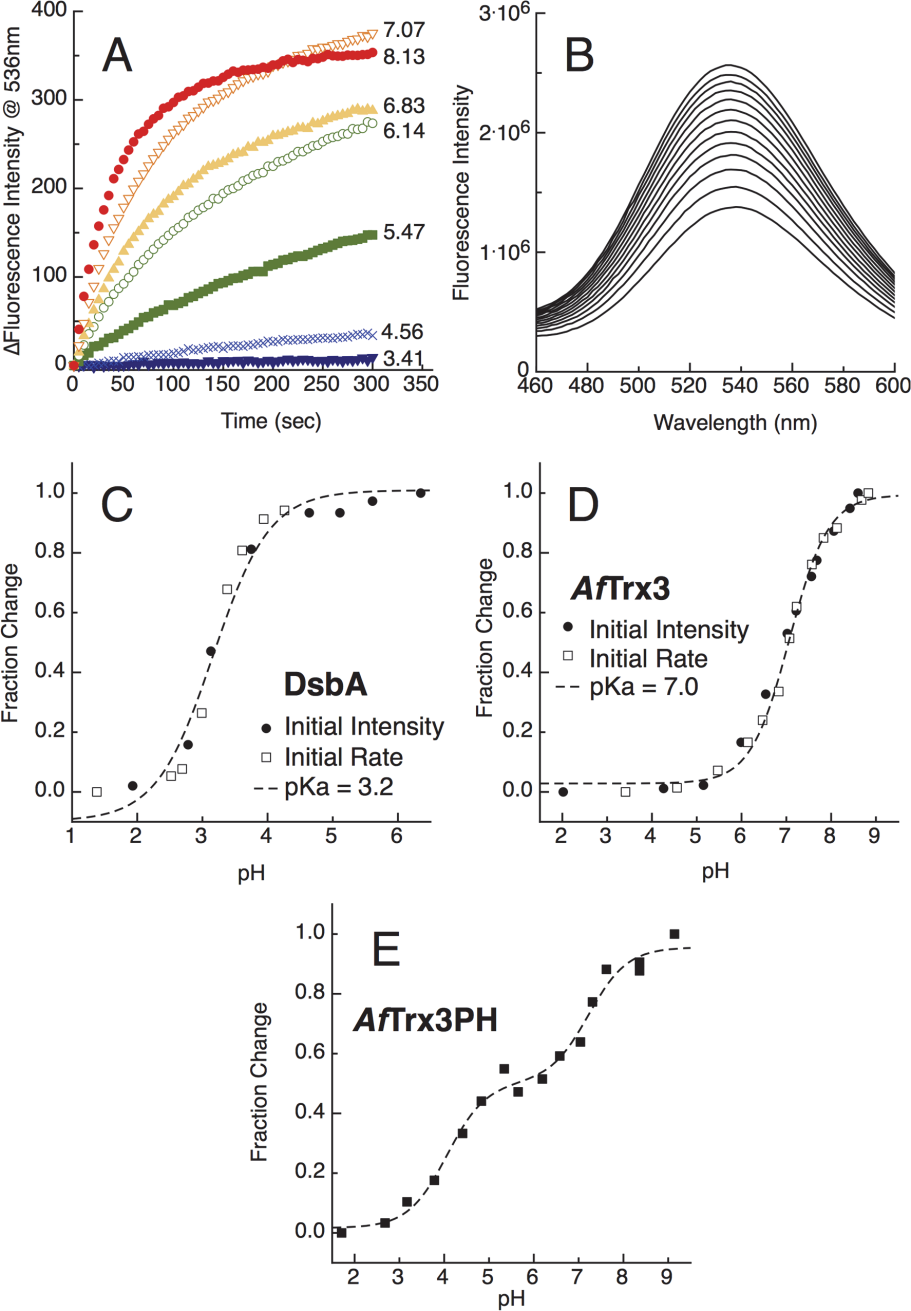
Cysteine thiol pKa determination. (A) The change in fluorescence intensity of *Af*Trx3 (0.25 μM) and BADAN (3.25 μM) over time in a variety of pH values. (B) The emission spectra of *Af*Trx3 (0.25 μM) and BADAN (3.25 μM) over time at pH 7.5, 387 nm excitation. Both the initial rates of the reaction, and the fluorescence intensity of equimolar protein and BADAN are then monitored as a function of pH to determine the spectroscopically monitored pKa values for: (C) DsbA, (D) *Af*Trx3, and (E) *Af*Trx3PH.

To support the finding by PFV that pK_a_ values are not correlated to the potentials of the Trx disulfide bonds studied here, we employed a spectroscopic method to verify the Cys pK_a_ values of the CXXC motif. The fluorescence probe BADAN was used to selectively label the thiolates of the CXXC [27] (**Fig 4**). Instead of monitoring the 240 nm optical absorbance of the thiolate itself [16], which we found to be unreliable, we chose the method developed by Lewin *et al*. [27,28] for its reproducibility. For these Trx proteins we monitored both the rate of the reaction with BADAN over time (**Fig 4A and 4B)**, as well as observed the initial fluorescence intensity of a reaction where equal amounts of protein and BADAN were mixed. Nearly identical results were found with each variation. Plotting either the initial velocity of the BADAN reaction, or the initial fluorescence intensity after mixing, as a function of pH, results in resolution of the spectroscopically determined pK_a_ values of the active site Cys residue(s), where DsbA is used as positive control (**Fig 4C**). The results are displayed in **Table 1**. Most of the Trx proteins were found to have a single pK_a_ of around 7, similar to *E*. *coli* Trx1. Importantly, the higher-potential Trx proteins (*Ta*Trx and *Af*Trx1) did not have low pK_a_ values. Interestingly, *Af*Trx3PH was found to have two pK_a_ values, one of which (4.2) has been shifted significantly (**Fig 4E**).

### The Energetics of Unfolding Resolves the Determinants of Redox Potential

**Fig 5:**
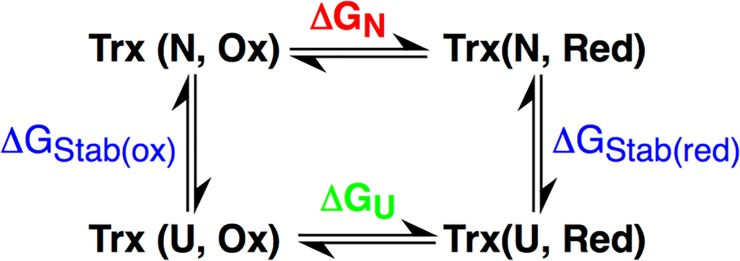
Thermodynamic square used to predict redox potential of disulfide bond containing proteins. Here, ΔG_N_ is determined and then correlated to a value of *E_m_*.

An alternate view of the control of disulfide reduction potentials has been ascribed to the difference in global folding stability of the Trx proteins themselves. In such a model, the redox potential of the natively folded protein can be calculated on the basis of a free-energy for the native redox reaction (ΔG_N_), which results from consideration of a thermodynamic square scheme (**Fig 5**). In such a model ΔG_N_ is derived from measurements of the folding/unfolding energetics of both the oxidized and reduced forms of each protein (which provide values of ΔG_Stab(Ox)_ and ΔG_Stab(Red)_) and a reference value of the redox potential of an “unfolded” protein-based disulfide bond (ΔG_U_).

**Fig 6:**
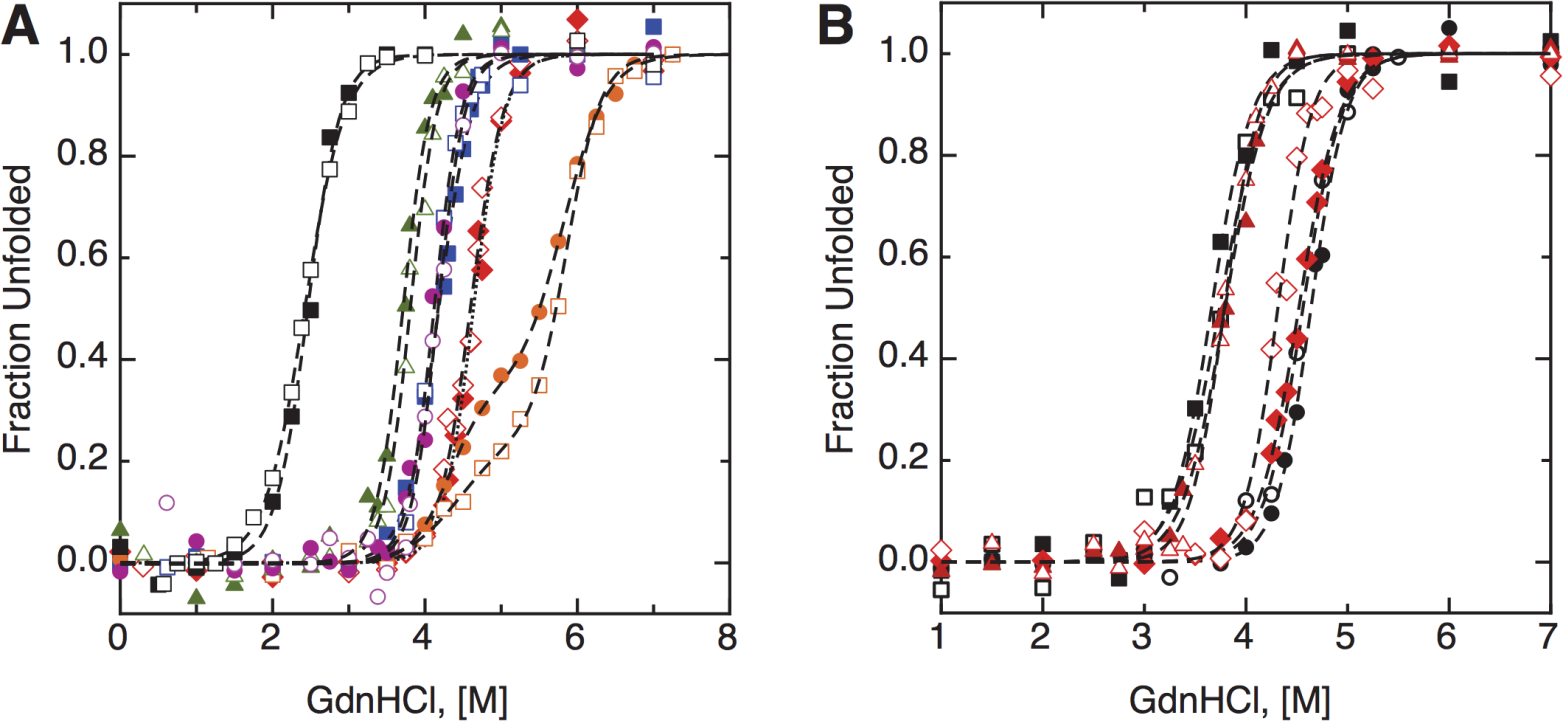
Fluorescence unfolding/refolding plots. The fraction of the protein that is unfolded is depicted versus the concentration of guanidine HCl in solution (A) closed symbols are unfolding and open symbols are refolding for *Af*Trx3/oxidized (red), *Af*Trx3PH/oxidized (blue), *Ta*Trx/oxidized (orange), *Af*Trx3/reduced (green), *Af*Trx3PH/reduced (pink), *Ta*Trx/reduced (black). For the sake of clarity, (B) shows similar curves for *Af*Trx3KP oxidized (black circles) and reduced (black squares), and *Af*Trx3HP oxidized (red diamonds) and reduced proteins (red triangles).

Folding and unfolding experiments were performed on the collection of *Af*Trx3 and the *Af*Trx3 mutants, and in each case the Trx revealed reversible, two-state unfolding (**Fig 6**). The oxidized forms of *Af*Trx3, *Af*Trx3HP and *Af*Trx3KP are more stable than the reduced forms, as determined by required concentrations of denaturant required to unfold the protein (D_1/2_) (**Table 2**). Having a more stable oxidized form is a trait of a reducing thioredoxin [6,37,38], and these results correlate to their midpoint potentials. For example, *Af*Trx3PH, has oxidized and reduced forms that are nearly equally stable, indicating the redox potential is higher than wild-type *Af*Trx3 (as found by PFV analysis). Free energies of stabilization at zero denaturant concentration (ΔG_Stab_), for the oxidized and reduced proteins were then calculated by extrapolating a linear dependence of free energy versus guanidinium concentration (see **S1 Methods** for equations [39,40]). Using these free energies and the square scheme of **Fig 5**, ΔG_N_ (and the corresponding redox potentials) can be found, though doing so requires a value of ΔG_U_. Here, we have used glutathione (*E*_*m*_ -240 mV[41]) as a reference point for an “unfolded” protein reduction potential [5,8,9] where ΔG_U_ is 7.9 kJ/mol [6]. Fundamentally this value is the redox potential of a disulfide bond in a chemical context similar to a peptide, and should be protein independent [8]. Indeed, using DsbA as a control, our results match what has been previously reported [6]. However, we note that through this method, we lack a genuine standard potential for an unfolded disulfide-bearing protein. Regardless, application of the thermodynamic square scheme allows for the generation of calculated *E*_*m*_ values for each Trx protein at pH 7, and **Table 2** provides a comparison of the thermodynamically calculated redox potentials with the experimental potentials listed in Table 1.

**Table 2:** Fluorescence unfolding/refolding thermodynamics and calculated midpoint potentials.

Trx	CXXC	D_1/2_ (Oxidized)	ΔG_Stab(Ox)_	D_1/2_ (Reduced)	ΔG_Stab(Red)_	Calculated *E*_*m*_
*Af*Trx3	CMPC	4.6 ± 0.2M	59 ± 3 kJ/mol	3.8 ± 0.1M	50 ± 6 kJ/mol	-245 mV
*Af*Trx3HP	CHPC	4.5 ± 0.1M	53 ± 2 kJ/mol	3.7 ± 0.1M	44 ± 2 kJ/mol	-246 mV
*Af*Trx3PH	CPHC	4.2 ± 0.1M	57 ± 6 kJ/mol	4.1 ± 0.1M	58 ± 2 kJ/mol	-194 mV
*Af*Trx3KP	CKPC	4.6 ± 0.2M	59 ± 1 kJ/mol	3.8 ± 0.1M	47 ± 5 kJ/mol	-261 mV
DsbA	CPHC	2.0 M	40 kJ/mol	2.1M	56 kJ/mol	-116 mV

*Af*Trx1 and *Ta*Trx folding experiments are complicated by the fact that these proteins likely contain one (*Af*Trx1) or two (*Ta*Trx) internal, structural disulfides (**S1 Fig**), yet only a single redox active disulfide detected by PFV. We believe the unfolding curves for the reduced versions of these proteins (*e*.*g*. see the black data for *Ta*Trx in **Fig 6A**) are shifted to lower guanidine concentrations due to the reduction of both the redox disulfide and the internal disulfides. The oxidized unfolding traces of *Ta*Trx do not show a clean two-state transition (orange data), including biphasic behavior as well as some degree of hysteresis in the refolding, which may be due to inter- or intra-protein disulfide bond exchange. Due to these complications, we focused on *Af*Trx3 and its mutants for the unfolding model studies. *Af*Trx3, *E*. *coli* Trx1 and DsbA each contain only the two cysteines that form the redox active disulfide, and follow a two-state unfolding. And their collective analyses clarify the potential impact of folding-based stabilization of disulfide bond redox potentials.

## Discussion

Here we report the first example of a full Pourbaix diagram for disulfide bounds of high potential found in thioredoxin proteins (*Af*Trx1 and *Ta*Trx). Through the examination of these and other archaeal Trx proteins, we have been able to specifically interrogate the pK_a_-governed model of control of disulfide-bond redox potentials. In all cases studied here, Pourbaix analysis (measuring complete pH-dependence of the potential) indicates a continuous slope of ~-60 mV/pH from low pH to above pH 9, suggesting 2H^+^:2e^-^ coupling. The electrochemical data themselves reveal that the redox couples must be for 2e^-^ processes due to the narrow peak-width at half-height values (**Table 1**). Complete descriptions of proton-coupled redox processes of disulfide bond-bearing proteins has been rare, particularly when considering the ubiquity of the Trx superfamily. And as noted above, previous work by Wunderlich and Glockshuber indicated with a small number of data-points that DsbA displays a pH-dependent potential of -49 mV/pH unit [5]. Although DsbA itself was found to be inactive to electrochemical analysis (due to insufficient adsorption at the electrodes utilized), here we have been able to use the rapid facility of PFV to our advantage, to produce more complete Pourbaix diagrams, with higher numbers of data points for other Trxs. Our approach allows us to fully describe the redox chemistry of higher-potential disulfide bonds found in the Trx family (*Af*Trx1 and *Ta*Trx), revealing that redox-linked protonations for the high-potential Trx proteins occur with pK_a_ values typical for Cys residues **(Fig 3B**), in disagreement with the pK_a_-governed model depicted in **Fig 1** [4].

In the pK_a_-governed redox model, the identity of the variable residues (and the inclusion of a His) in the CXXC is thought to impact the redox potential. Experimentally we have examined this issue with the *Af*Trx3 protein, using it as a scaffold to produce *Af*Trx3HP (CHPC) and *Af*Trx3PH (CPHC) mutants. The *Af*Trx3HP mutant behaves similarly to the wild-type (CMPC) protein: The midpoint potential is low, and the unfolding/refolding data does not show a major deviation from wild-type. *Af*Trx3PH, however, is different from wild-type. This mutant has a +66 mV shift in the redox potential, but again reveals Pourbaix diagrams devoid of features indicating a uniquely low pK_a_ associated with redox chemistry. The unfolding/refolding data for *Af*Trx3PH also differs from wild-type, as both the oxidized and reduced forms are equally stable. Notably, using the spectroscopic probe BADAN, we determined the apparent pK_a_ for Cys residues. By the spectroscopic approach, the placement of His in the second variable position (like DsbA) does result in a shifted pK_a_ value (4.2, Fig 4E). Yet these values do not correlate to redox-linked pK_a_ values as determined by direct electrochemistry, nor do they correlate with redox potentials. Thus, we find that the spectroscopically reported values may result from aggregate phenomena that are not linked to redox chemistry directly (as is portrayed by a Pourbaix diagram).

In addition to the specific role in modulation of Cys pK_a_ values, hydrogen-bonding interactions (particularly with the N-terminal Cys residue) have been implicated in the activity of Trx proteins by Berndt and co-workers [42]. Intriguingly, here there are no apparent hydrogen-bonding interactions at work in the Trx active site, as observed for the *Af*Trx3HP structure. Indeed the disulfide is in a hydrophobic environment, blocked from direct contact with solvent by residues Trp 58, Pro 61, Ile 102, Pro 103. The only potential hydrogen bond is from the N-term Cys to the carbonyl of Ile 102 (3.18 Å). There are no H-bonding networks that would be expected to alter the pKa of either Cys. In fact, both cysteine side chains are beautifully sandwiched by hydrophobic residues on all sides.

Might there be any role for histidine in the determination of the redox potentials of Trx-based disulfide bonds? Inspection of the structure of the *Af*Trx3HP CHPC motif (**Fig 2**, inset) shows M60H is oriented out into solution, and is neither able to interact with the cysteine in the active site nor is it able to provide any stabilizing hydrogen bonding interactions with any part of the protein fold. Thus, our structure is consistent with the data presented above and helps to explain why the M60H mutation has no influence on either the redox or folding properties of *Af*Trx; this histidine sidechain is making no contacts. Although a structure of *Ta*Trx is not available, we predict that the histidine of the CHPC motif will also point out into solution based on the *Af*Trx3HP structure, which is the first of a Trx with a CHPC motif. Therefore, it seems unlikely that the histidine of the CHPC motif will be at all responsible for the high redox potential of *Ta*Trx. Although having a histidine at position 2 does not appear to have an influence on the redox properties, we also considered the relative importance of histidine at position 3. *Af*Trx3PH places histidine at position 3, with a sequence motif identical to DsbA (CPHC), yet the redox properties are more similar to wild-type *Af*Trx3 (-221 mV and -278 mV, respectively) than there are to DsbA (-89, -124 mV), even though one pK_a_ is observed spectroscopically at 4.2, and without structural evidence, we can only presume that the His present in *Af*Trx3PH could engage in hydrogen-bonding interactions with one or both of the redox-active cysteines. Again, the spectroscopic determinations of the Cys pK_a_s do not correlate with the direct observation of redox-linkage observed in a Pourbaix diagram. Here we can conclude that in terms of redox-reactivity, the cysteines of the Trx proteins considered here are more like typical Cys side-chains, while the fluorescence-detected pK_a_ values likely report on aggregated phenomena that is not tied to redox chemistry (and aside from the *Af*Trx3PH protein, may report on the composite pK_a_s of both Cys residues). Considering all of the data in **Table 1**, there seems to be little correlation between CXHC proteins and potential, but perhaps the greater role in tuning redox potential is due placement of the proline residue, suggesting that overall or local folding traits more profoundly impact the redox potential.

**Fig 7:**
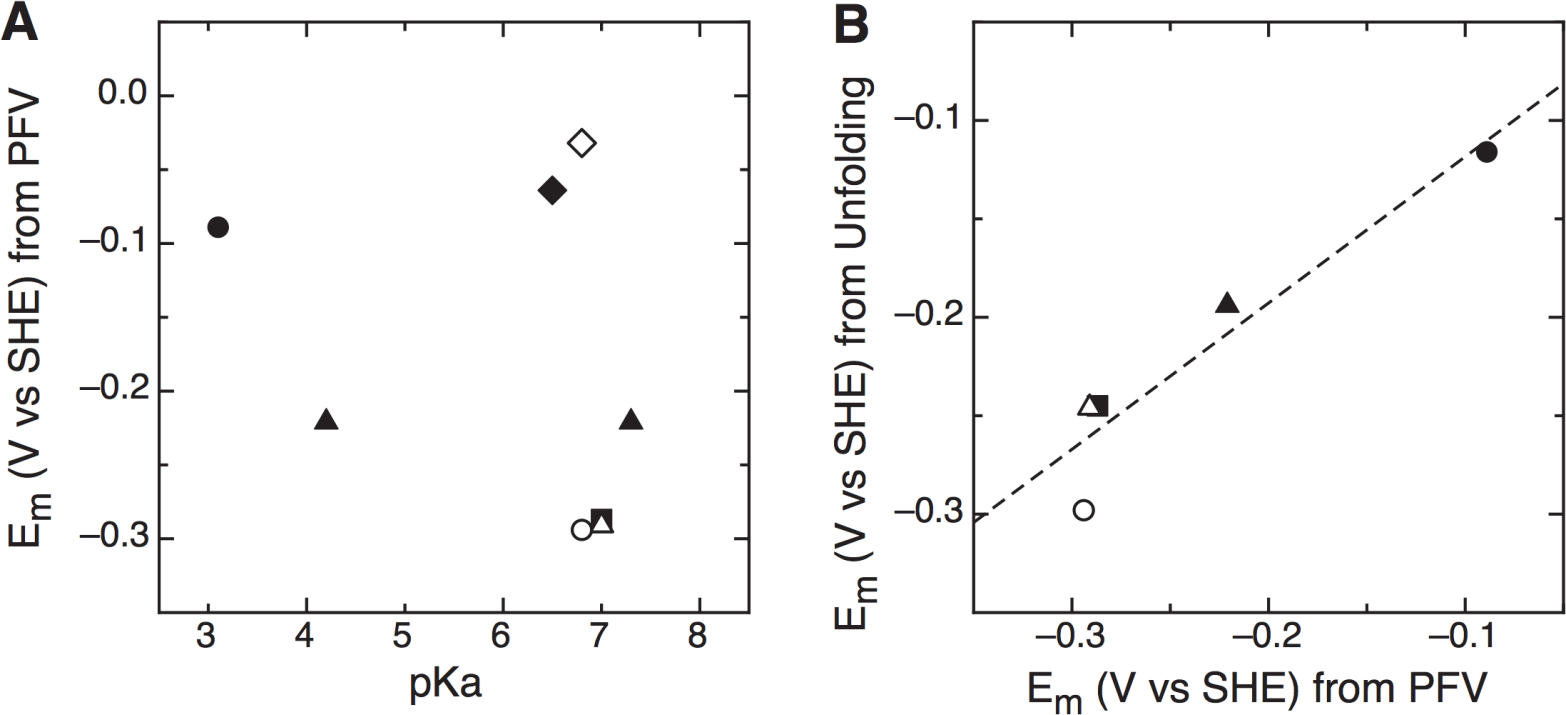
Correlations (and lack thereof) between redox potentials and other factors. (A) The pK_a_-governed model cannot resolve a prediction of midpoint potential (*Ta*Trx (closed diamond), *Af*Trx1 (closed diamond), *Af*Trx3 (closed square), *Af*Trx3HP (open triangle), *Af*Trx3PH (closed triangle), *Af*Trx3KP (open circle), DsbA (closed circle)). (B) Global folding energies, used to calculate an *E_m_* value correlate with potentials directly determined by PFV (*Af*Trx3 (closed square), *Af*Trx3HP (open triangle), *Af*Trx3PH (closed triangle), *Af*Trx3KP (open circle), DsbA (closed circle)).

Overall our data show that the solution-based pK_a_ values do not govern the redox properties displayed by the Trx disulfide bonds. In solution, the pK_a_ values of the free thiolates can only be measured when the protein is already reduced. These solution assays do not directly probe what the protonation state is *while* the reduction is occurring, in contrast to PFV. Thus, while the spectroscopically determined pK_a_ values appear to be somewhat dependent on the nature of the CXXC motif, they do not correlated with the redox event that reduces the disulfide (**Fig 7A**). Aside from the pK_a_ contribution (or lack thereof) our data show that the overall folding thermodynamics, when two-state folding can be applied, does quantitatively predict redox reactivity (**Fig 4** and **Table 2**). Whether or not this relationship is causal, cannot be determined at this time, yet it is clear that there is a correlation (**Fig 7B**). Structurally, *Af*Trx3, *Ec*Trx and others are similar, even if their CXXC differs, suggesting once again that the pK_a_ alone is not driving the midpoint potential, and therefore reactivity. Given the roles that Trx paralogs and Trx domains play in protein:protein redox reactions, a long-term ramification of this work is that intermolecular interactions may further modulate protein structure to tune the redox characteristics and reactivity of Trx disulfide bonds. We note as well that the thermodynamic potential alone may not govern the reactivity of any disulfide bond, particularly *in vivo*. For example, *E*. *coli* glutaredoxin 1 has a higher potential disulfide bond (-233 mV) than the principle Trx protein (-270 mV) [8], yet the differences in *K*_*m*_ for these proteins results in Grx1 being a better reducing system for ribonucleotide reductase, as measured by *k*_*cat*_ */K*_*m*_ [43].

## Conclusion

Here we show that when the electrochemistry of thioredoxins from archaea is directly probed, both the high and low potential thioredoxins behave similarly. Our Pourbaix diagrams show slopes of -60 mV/pH around neutral pH suggesting the solution-based pK_a_ values are not accurate representations of the proton coupled electron transfer event. These results call into question the validity of the previously proposed pK_a_ control model and show it is not a good fit for these archaeal thioredoxins. Additionally, however, we show that the protein fold and stability of the oxidized and reduced forms of the protein can predict whether a redox potential will be oxidizing or reducing.

## Supporting Information

S1 Fig Sequence alignment of *E. coli* Trx1, *T*. *acidophilum* Trx and *A*. *fulgidus* Trx1 and Trx3 using sequences deposited at NCBI and Clustal 2.1 for the alignment. Additional Cys residues are highlighted in teal.
(PDF)

S1 Methods Additional fitting routines for pKa fitting routines and folding/unfolding experiments. (PDF)

S1 Table List of mutagenesis primers used to make *Archaeoglobus fulgidus* Trx3 mutants. (PDF)

S2 Table Data collection and refinement statistics for *AfTrx3HP*. ^a^Values in parentheses are the highest resolution shell. ^b^ *R*_*sym*_ = ∑_*hkl*_∑_i_|*I*_*i*_(*hkl*)-<*I*(*hkl*)>|/∑_hkl_∑_i_ *I*_*i*_(*hkl*), where *I*_*i*_(*hkl*) is the i^th^ measured diffraction intensity and <*I*(*hkl*)> is the mean intensity for the reflection with the miller index (hkl). ^c^ *R*_*work*_ = ∑_hkl_||*F*_*o*_(*hkl*)|-|*F*_*c*_(*hkl*)||/∑_hkl_|*F*_*o*_(*hkl*)|. ^d^ *R*_f*ree*_ = *R*_work_ for 5% of reflections omitted from refinement. ^e^asu, asymmetric unit. ^f^RMS, root mean square.
(PDF)
