# Supplementary material for: Rheostat Re-Wired: Alternative Hypotheses for the Control of Thioredoxin Reduction Potentials
Source: PLoS One. 2015 Apr 13;10(4):e0122466. doi: 10.1371/journal.pone.0122466 (PMC4395160; doi:10.1371/journal.pone.0122466)
Supplement: S1 Fig — Additional Cys residues are highlighted in teal. (PDF) [file pone.0122466.s001.pdf]

```
EcTrx1          MLYESVSGEIKSTFSWLMLHQQRNQHARLIPVELYMSDKIIHLTDDSFDTDVLKADGAIL 60
AfTrx3          --MDELELIRQKKLKEMMQKMSGEEKARKV------LDSPVKLNSSNFD-ETLKNNENVV 51
TaTrx           -----------------MKNYMG---------------CVKDITFNDFN-RLIDEKKSFI 27
AfTrx1          ------MPMVRKAAFYAIAVISG-----------VLAAVVGNALYHNFN--SDLGAQAKI 41
                                 :                       .    .*:          :

EcTrx1          VDFWAEWCGPCKMIAPILDEIADEYQGKLTVAKLNIDQNPGTAPKYGIRGIPTLLLFKNG 120
AfTrx3          VDFWAEWCMPCKMIAPVIEELAKEYAGKVVFGKLNTDENPTIAARYGISAIPTLIFFKKG 111
TaTrx           IELWADWCHPCKIMAPYLEEACQKLN-ACYFYKINIDENPEIVDTLNVNSIPRIIMFVEG 86
AfTrx1          YFFYSDSCPHCREVKPYVEEFAKTHNLTWCNVAEMDANCSKIAQEFGIKYVPTLVIMD-- 99
                  :::: *  *: : * ::* ..              : .  .   .:  :* ::::

EcTrx1          EVAATKVGALSKGQLKEFLDANLA--- 144
AfTrx3          KPVDQLVGAMPKSELKRWVQRNL---- 134
TaTrx           QRQAELKGFQKLQSIIDQISKIPCDSV 113
AfTrx1          EEAHVFVGSDEVRTAIEGMK------- 119
                :      *          :.
```

Figure S1. Sequence alignment of *E. coli* Trx1, *T. acidophilum* Trx and *A. fulgidus* Trx1 and Trx3 using sequences deposited at NCBI and Clustal 2.1 for the alignment. Additional Cys residues are highlighted in teal.
