# Supplementary material for: Rheostat Re-Wired: Alternative Hypotheses for the Control of Thioredoxin Reduction Potentials
Source: PLoS One. 2015 Apr 13;10(4):e0122466. doi: 10.1371/journal.pone.0122466 (PMC4395160; doi:10.1371/journal.pone.0122466)
Supplement: S1 Methods — (PDF) [file pone.0122466.s002.pdf]

SUPPORTING INFORMATION for Bewley KD, *et al.*

Rheostat rewired: alternative hypotheses for the control of thioredoxin reduction potentials

SUPPORTING METHODS

Data Fitting for pK_a Determination

Data were fit to either a single or double pK_a model described by:

For one pK_a: $$y = \frac{ASH + AS \times 10^{pH-pKa}}{1 + 10^{pH-pKa}}$$

y = observed fluorescence intensity or rate
ASH = fluorescence intensity or rate of the protonated form of the protein
AS = fluorescence intensity or rate of the deprotonated form of the protein

For two pK_a's: $$y = \frac{ASHH + ASH \times 10^{pH-pKa1} + AS \times 10^{2pH-pKa1-pKa2}}{1 + 10^{pH-pKa1} + 10^{2pH-pKa1-pKa2}}$$

y = observed fluorescence intensity or rate
ASHH = fluorescence intensity or rate of the protonated form of the protein
ASH = fluorescence intensity or rate of the protein with one proton
AS = fluorescence intensity or rate of the deprotonated form of the protein

Data Fitting for Protein Unfolding/Refolding

Data were fit to either a two-state model, or a biphasic model in the case of *Af*Trx3d, as follows:

For a two-state model: $$y = \frac{y_N + y_U \times \exp^{(m[D]-\Delta G)/RT}}{1 + \exp^{(m[D]-\Delta G)/RT}}$$

y = observed fluorescence signal
y_N = signal due to folded protein
y_U = signal due to unfolded protein
m = describes the dependence of ΔG on denaturant concentration
[D] = denaturant concentration
ΔG = free energy change of unfolding
R = gas constant
T = temperature (°K)

Biphasic unfolding : $$y = \frac{y_N + y_I \times \exp^{(m_1[D]-\Delta G_1)/RT} + y_U \times \exp^{(m_1[D]-\Delta G_1+m_2[D]-\Delta G_2)/RT}}{1 + \exp^{(m_1[D]-\Delta G_1)/RT} + \exp^{(m_1[D]-\Delta G_1+m_2[D]-\Delta G_2)/RT}}$$

y_I = signal due to the intermediate unfolding state
$m_1/\Delta G_1$ = refers to the first transition
$m_2/\Delta G_2$ = refers to the second transition
