# Supplementary material for: Rheostat Re-Wired: Alternative Hypotheses for the Control of Thioredoxin Reduction Potentials
Source: PLoS One. 2015 Apr 13;10(4):e0122466. doi: 10.1371/journal.pone.0122466 (PMC4395160; doi:10.1371/journal.pone.0122466)
Supplement: S1 Table — (PDF) [file pone.0122466.s003.pdf]

Table S1. List of mutagenesis primers used to make *Archaeoglobus fulgidus* Trx3 mutants.

Mutant	Primers
CHPC<br>(*Af*Trx3s)	For 5'- C TGG GCG GAG TGG TGC CAC CCG TGC AAA ATG ATT GC -3'<br>Rev 5'- GC AAT CAT TTT GCA CGG GTG GCA CCA CTC CGC CCA G -3'
CPHC<br>(*Af*Trx3d)	For 5'- C TGG GCG GAG TGG TGC CCG CAC TGC AAA ATG ATT GC -3'<br>Rev 5'- GC AAT CAT TTT GCA GTG CGG GCA CCA CTC CGC CCA G -3'
CKPC<br>(*Af*Trx3K)	For 5'- C TGG GCG GAG TGG TGC AAG CCG TGC AAA ATG ATT GC -3'<br>Rev 5'- GC AAT CAT TTT GCA CGG CTT GCA CCA CTC CGC CCA G -3'
