# Supplementary material for: Rheostat Re-Wired: Alternative Hypotheses for the Control of Thioredoxin Reduction Potentials
Source: PLoS One. 2015 Apr 13;10(4):e0122466. doi: 10.1371/journal.pone.0122466 (PMC4395160; doi:10.1371/journal.pone.0122466)
Supplement: S2 Table — aValues in parentheses are the highest resolution shell. b R sym = ∑hkl∑i|I i(hkl)-<I(hkl)>|/∑hkl∑i I i(hkl), where I i(hkl) is the ith measured diffraction intensity and <I(hkl)> is the mean intensity for the reflection with the miller index (hkl). c R work = ∑hkl||F o(hkl)|-|F c(hkl)||/∑hkl|F o(hkl)|. d R free = R work for 5% of reflections omitted from refinement. easu, asymmetric unit. fRMS, root mean square. (PDF) [file pone.0122466.s004.pdf]

**Data Collection**	
Space Group	$P2_12_12_1$
Unit Cell Dimensions	
a (Å)	46.56
b (Å)	59.91
c (Å)	100.08
Beam line	R-axis IV
Wavelength (Å)	1.54
Resolution Range (Å)	50-1.95 (2.02-1.95)
Observations	73689
Unique Reflections	18906
Redundancy	3.9
Completeness (%)[a]	88.4 (99.7)
$I/\sigma(I)$[a]	25.42 (5.25)
R_{sym}[b] (%)[a]	5.2 (23.2)
**Refinement**	
R_{work}[c] (R_{free}[d]) (%)	19.07 (23.57)
Resolution Range (Å)	50-1.95
No. Reflections	18765
No. Molecules per asu[e]	2
No. of non-hydrogen atoms:	
Protein	1766
Water	328
Average B-Factors ($Å^2$)	
Protein atoms	25.2
Water	25.9
RMS Deviations[f]:	
Bond Lengths(Å)	0.007
Bond Angles(°)	0.9
Ramachandran Plot (%)	
most favored	93.7
additionally allowed	6.3
generously allowed	-
disallowed	-

Table S2. Data collection and refinement statistics for *Af*Trx3s. [a]Values in parentheses are the highest resolution shell. [b]$R_{sym}=\sum_{hkl}\sum_i|I_i(hkl)-\langle I(hkl)\rangle|/\sum_{hkl}\sum_i I_i(hkl)$, where $I_i(hkl)$ is the ith measured diffraction intensity and $\langle I(hkl)\rangle$ is the mean intensity for the reflection with the miller index (hkl). [c]$R_{work}=\sum_{hkl}||F_o(hkl)|-|F_c(hkl)||/\sum_{hkl}|F_o(hkl)|$. [d]$R_{free}=R_{work}$ for 5% of reflections omitted from refinement. [e]asu, asymmetric unit. [f]RMS, root mean square
